# Emerging Parasitic Protists: The Case of Perkinsea

**DOI:** 10.3389/fmicb.2021.735815

**Published:** 2022-01-13

**Authors:** Sarah Itoïz, Sebastian Metz, Evelyne Derelle, Albert Reñé, Esther Garcés, David Bass, Philippe Soudant, Aurélie Chambouvet

**Affiliations:** ^1^Univ Brest, CNRS, IRD, Ifremer, LEMAR, Plouzané, France; ^2^Departament de Biologia Marina i Oceanografia, Institut de Ciències del Mar, CSIC, Pg. Marítim de la Barceloneta, Barcelona, Spain; ^3^Centre for Environment, Fisheries and Aquaculture Science (Cefas), Weymouth, United Kingdom; ^4^Department of Life Sciences, The Natural History Museum, London, United Kingdom; ^5^Biosciences, University of Exeter, Exeter, United Kingdom; ^6^Sorbonne Université, CNRS, UMR 7144 Adaptation et Diversité en Milieu Marin, Ecology of Marine Plankton (ECOMAP), Station Biologique de Roscoff SBR, Roscoff, France

**Keywords:** *Perkinsus*, Parvilucifera, X-cell parasite, broad host range parasite, emerging diseases, severe Perkinsea infection, opportunistic parasite

## Abstract

The last century has witnessed an increasing rate of new disease emergence across the world leading to permanent loss of biodiversity. Perkinsea is a microeukaryotic parasitic phylum composed of four main lineages of parasitic protists with broad host ranges. Some of them represent major ecological and economical threats because of their geographically invasive ability and pathogenicity (leading to mortality events). In marine environments, three lineages are currently described, the Parviluciferaceae, the Perkinsidae, and the Xcellidae, infecting, respectively, dinoflagellates, mollusks, and fish. In contrast, only one lineage is officially described in freshwater environments: the severe Perkinsea infectious agent infecting frog tadpoles. The advent of high-throughput sequencing methods, mainly based on 18S rRNA assays, showed that Perkinsea is far more diverse than the previously four described lineages especially in freshwater environments. Indeed, some lineages could be parasites of green microalgae, but a formal nature of the interaction needs to be explored. Hence, to date, most of the newly described aquatic clusters are only defined by their environmental sequences and are still not (yet) associated with any host. The unveiling of this microbial black box presents a multitude of research challenges to understand their ecological roles and ultimately to prevent their most negative impacts. This review summarizes the biological and ecological traits of Perkinsea—their diversity, life cycle, host preferences, pathogenicity, and highlights their diversity and ubiquity in association with a wide range of hosts.

## Introduction

Parasitism is a key component in all ecosystems, playing a fundamental role at the population level and wider ecological scales. Although parasites play a key role in food web interactions (Bjorbækmo et al., 2020), their diversity, dynamics, and influence on ecosystems remain neglected (Lafferty et al., 2006). In marine ecosystems, global environmental sequencing studies, based on the analysis of the small subunit rRNA-encoding gene (SSU rDNA) have revolutionized our conception of the microbial food webs with the (re-)discovery of an undescribed diversity shaping a more complex and cryptic global interactive network (Lima-Mendez et al., 2015; Guidi et al., 2016). However, the vast majority of these putative parasitic organisms are still only identified by taxonomic marker gene sequences and fundamental questions about host preference, range, and interactions remain unanswered.

**FIGURE 1:**
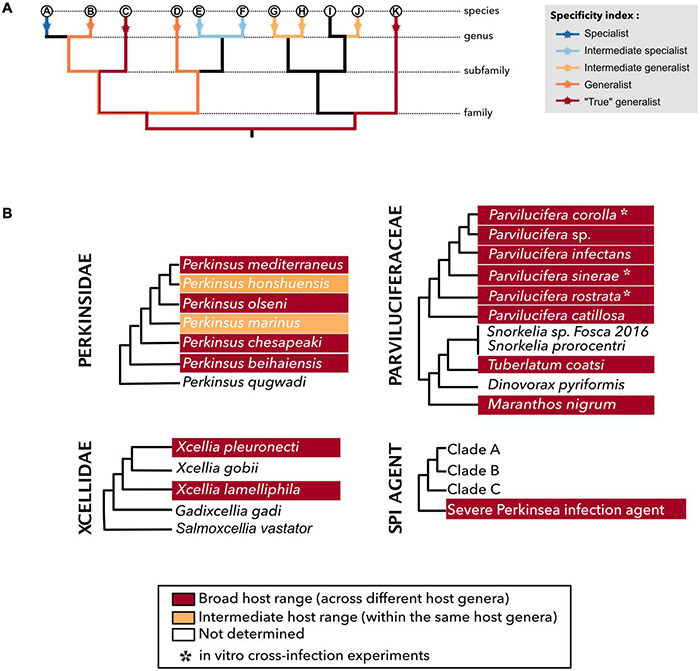
Specificity index within the Perkinsea members. **(A)** Class of specificity index adapted from Desdevises et al. (2002) and Šimková et al. (2006). Specialists and intermediate specialists are considered as narrow host range (NHR), whereas intermediate generalists, generalists, and “true” generalists are considered as broad host range (BHR). **(B)** Schematic phylogenies of Parviluciferaceae, Perkinsidae, Xcellidae, and SPI agent where each species was affiliated to a specificity index class (see Supplementary Table 1 for more details).

Parasitic organisms are defined on the basis of their trophic characteristics and, therefore, by their host range, which allow or not the spread to a novel host species or into new biogeographical areas (Cleaveland et al., 2001; Poulin and Mouillot, 2003). The host range, defined by Lymbery (1989), considers the number of host species by a given parasite species. Two main categories can be distinguished by the extent diversity of hosts infected: the narrow host range (NHR) and the broad host range (BHR). Despite NHR parasites seem to slightly dominate the host–symbiont networks as described by Bjorbækmo et al. (2020), this dichotomy between BHR and NHR parasites is not static, and events like host shift can lead NHR to become BHR parasites and vice versa (Agosta et al., 2010). The distinction between NHR and BHR does not seem to be clear-cut for the majority of the parasitic taxa. Therefore, Desdevises et al. (2002) developed the non-specific index (NSI), which classifies parasites based on their host range and the relatedness between host organisms. This index was adapted by Šimková et al. (2006) as the specificity index (SI) based on cyprinid fish and *Dactylogyrus* spp. (monogenean gill flukes) model with the following categories: (1) strict specialist living on a single host species, (2) intermediate specialist living on two or more host species from the same genus, (3) intermediate generalist living on two or more non-congeneric species of the same terminal clade, (4) generalist living on different hosts belonging to one host family and (5) “true” generalist living on different host species of different families (Figure 1A). However, estimating host range is particularly challenging in the case of uncultivable parasitic microorganisms. Recent studies highlight the strong potential of molecular methods, such as PCR, to detect putative cryptic host–parasite associations [see review by Bass et al. (2015)]. However, these molecular methodologies only reflect the presence/absence of the genetic signature of the parasite within the host tissue and do not identify paratenic, reservoir, dead-end, or accidental hosts. Hence, the combination of molecular and microscopic techniques should provide a powerful tool to access the putative range of host–parasite associations. In this review, to avoid confusion, we will look on how the putative association between hosts and parasites has been estimated by focusing, if possible, on the combination of molecular and microscopic detection methodologies. Finally, we consider here the host–parasite association in terms of species richness following the SI, but also in terms of accumulated scientific knowledge on these host–parasite interactions and choose to define BHR parasite as an organism able to ensure its own survival through the colonization of divergent host species in the same ecosystem or following a translocation event.

In recent years, host–parasite interactions have been influenced by anthropogenic consequences (e.g., global warming, habitat degradation, mass extinction, and introduction of exotic host or parasitic species). This phenomenon is illustrated by parasitic species dissemination worldwide and the severity of infectious disease in time and space, which can lead to mortality events (Harvell, 1999; Daszak, 2000). Indeed, interactions between a host and its pathogens are not fixed in time and space. They depend both on the efficiency of the defense of the host (immune system or strategies) and on mechanisms used by parasites to bypass these defense systems to establish infection (Råberg et al., 2014). Indeed, environmental stressors (e.g., temperature, pollution) modify the immune system of the host, which increase their vulnerability to parasitism (Goedken et al., 2005; Morley, 2010). For example, in a metazoan host, *P. marinus*-infected oysters exposed to the antifouling agent tributyltin (TBT) showed an increase in prevalence and mortality (Anderson et al., 1996), or in a protist host, herbicide exposure could lead to increase in chytrid infections in phytoplankton populations (e.g., Van den Wyngaert et al., 2013).

In addition, the introduction of exotic species, described as a global phenomenon, is also considered as a main factor of alteration of local networks (Cohen, 1998; Galil, 2000; Goedken et al., 2005). Over the last two centuries, 37% of the first recorded species introduction phenomenon occurred in the last 50 years (1970–2014), and this trend is not slowing down (Seebens et al., 2017). In aquatic ecosystems, the unintentional introduction of exotic species has increased substantially with the globalization of economies: *Haplosporidium nelsoni* (hypothetic Livestock vector: *Crassostrea gigas*) translocated from Asia to the United States (USA) (Burreson et al., 2000) or *Bonamia ostreae* (hypothetic livestock vector: *Ostrea edulis*) translocated from the United States to Europe (Elston et al., 1986). Each day, hundreds of species are passively transferred across the oceans by sea transport (e.g., ballast water), pet trade, or stock exchange of organisms (Ruiz et al., 2000; Patoka et al., 2018). If the majority of pest introductions fail due to the hostility of the new ecosystem (Williamson and Fitter, 1996; de Montaudouin et al., 2001), some find a new niche for their development and reproduction (e.g., the absence of enemies, such as predators, parasites, and competitors). Moreover, parasitic microorganisms with a high potential to invade and quickly adapt to a new environment share several common traits as being *r*-strategist, a wide host range with high phenotypic plasticity, high dispersal capacities and genetic diversity (Sakai et al., 2001; Litchman, 2010). All these conditions form the “ecological roulette” described by Cariton and Geller (1993).

Hence, the introduction of exotic parasites could lead to the emergence of new pathologies with, in some cases, disastrous consequences for the local host populations. One of the most famous examples of an emerging disease is the “Dermo” disease caused by the virulent and invasive protist *Perkinsus marinus* (Perkinsea, Alveolata). This parasitic protist has been identified as responsible for the mortality events of oysters (*Crassostrea virginica*) in, for example, the Gulf of Mexico and the Chesapeake Bay (United States) (Burreson et al., 1994; Andrews, 1996). This microeukaryote belongs to the Perkinsea (syn. Perkinsids, Perkinsozoa) lineage Alveolata, which has long been ignored in aquatic ecosystems with the exception of the notifiable agents, *P. olseni* and *P. marinus*. Nowadays, four parasitic lineages of Perkinsea have been described from a wide variety of aquatic ecosystems: Perkinsidae, Parviluciferaceae, Xcellidae, and the severe Perkinsea infection (SPI) agent.

Due to a general lack of knowledge of the diversity and biology of these parasitic protists, our understanding of their impact on the structure and functioning of aquatic ecosystems remains limited. Our objective here is to review the main discovery of described Perkinsea lineage, of which some representative organisms could be classified as putative BHR species with a strong capacity to become successful invasive species in a context of global change.

### *Perkinsus* spp., an Emerging Parasite of Mollusks

**FIGURE 2:**
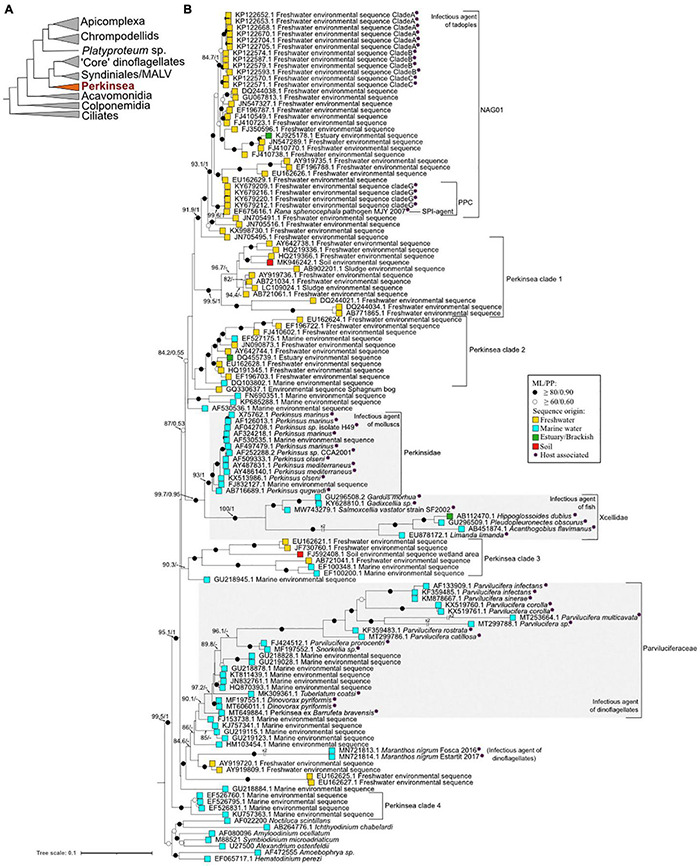
Major phylogenetic relationships within the Perkinsea and other alveolates. **(A)** Schematic radiation of Alveolata superphylum (not to scale) based on their rDNA phylogeny adapted from Chambouvet et al. (2020). The basal branch (dotted line) is hypothetical. **(B)** Maximum likelihood tree investigating the Perkinsea diversity based on 18S rRNA. The phylogeny was calculated from 134 taxa and 1,431 character alignment position. Seven sequences of dinoflagellates, Syndiniales, and MALV (marine alveolate) were used as an outgroup. ML bootstrap values (1,000 replicates, GTR+F+R5) and Bayesian posterior probability (8,000,000 generations, GTR+G+R5 model) were notated using the following convention: support values are summarized by black circles when ≥ 80%/0.9 and white circles when it is not the case but values ≥ 60%/0.6. When the topology is inconsistent in one of the inference methods, it is denoted by a ‘−’ (see phylogenetic analysis details in Supplementary SMM). The sequence origins are represented by squares of colors: light blue for marine waters, yellow for land waters, green for brackish waters, and red for wetland soil. Purple asterisks indicate host-associated sequences. The tree was annotated using Interactive Tree of Life (IToL) (https://itol.embl.de/, Letunic and Bork, 2019) and Inkscape (https://inkscape.org/en/).

In 1946, following a mass mortality event (MME) of oyster stocks in Louisiana (Gulf of Mexico, United States), Mackin et al. (1950) identified a protist present in host tissue sample as the causative agent. First affiliated to a fungal lineage and named *Dermocystidium marinum* (Mackin et al., 1950), electronic microscopy observations then showed morphological characteristics, such as the presence of a subpellicular membrane, micropores, and a conoid-like structure (zoospore stage) suggesting a re-affiliation of this protist into the Apicomplexa phylum (Alveolata) (Perkins and Menzel, 1967; Perkins, 1976). While doubts remained about the accuracy of this affiliation (Siddall et al., 1997), molecular phylogenies based on ribosomal and actin sequences revealed that *Perkinsus* species were more closely related to dinoflagellates than apicomplexans (Reece et al., 1997). In 2003, multiple protein phylogeny indicated that *Perkinsus* is an early branch in dinoflagellate lineage (see schematic representation in Figure 2A; Saldarriaga et al., 2003).

Nowadays, seven species are described within the genus *Perkinsus*: *P. marinus*, *P. olseni*, *P. qugwadi*, *P. chesapeaki*, *P. mediterraneus*, *P. honshuensis*, and *P. beihaiensis* (see Figures 1B, 2B for the phylogenetic classification of these parasites). However, only *P. marinus* and *P. olseni*, the etiological agents of “Dermo” disease and Perkinsosis, respectively, have significant negative impacts on mollusk populations worldwide (Siddall et al., 1997). These two infectious agents are today within the list of notifiable diseases in the World Organization for Animal Health (OIE) (Oie-Listed Diseases, 2021: OIE—World Organisation for Animal Health).

Some *Perkinsus* species have a putative BHR among mollusk species as, for example *P. olseni* infecting various clams (e.g., *R. decussatus*, *R. philippinarum*, *Austrovenus stutchburyi*, *Tridacna maxima*, *T. crocea*, *and Pitar rostrata*), oysters (*Crassostrea rhizophorae*, *Saccostrea* sp.), pearl oyster (e.g., *Pinctada imbricata* and *P. fucata*), and abalone (e.g., *Haliotis rubra* and *H. laevigata*) (Lester and Davis, 1981; Cremonte et al., 2005; Dungan et al., 2007; Sheppard and Phillips, 2008; Sanil et al., 2010; Ramilo et al., 2015; Pagenkopp Lohan et al., 2018), or *P. chesapeaki*, which can infect clams (*R. decussatus*, *R. philippinarum*) and oysters (*C. rhizophorae*, *C. virginica*) (Coss et al., 2001; Arzul et al., 2012; Dantas Neto et al., 2016). Conversely other *Perkinsus* species show an intermediate host range as, for example, *P. marinus* infecting mostly oysters (e.g., *C. agar*, *C. rhizophorae*, *C. virginica*, *C. agar*, and *Saccostrea palmula*) (e.g., Enríquez-Espinoza et al., 2010; Cáceres-Martínez et al., 2012; da Silva et al., 2013, 2014).

**FIGURE 3:**
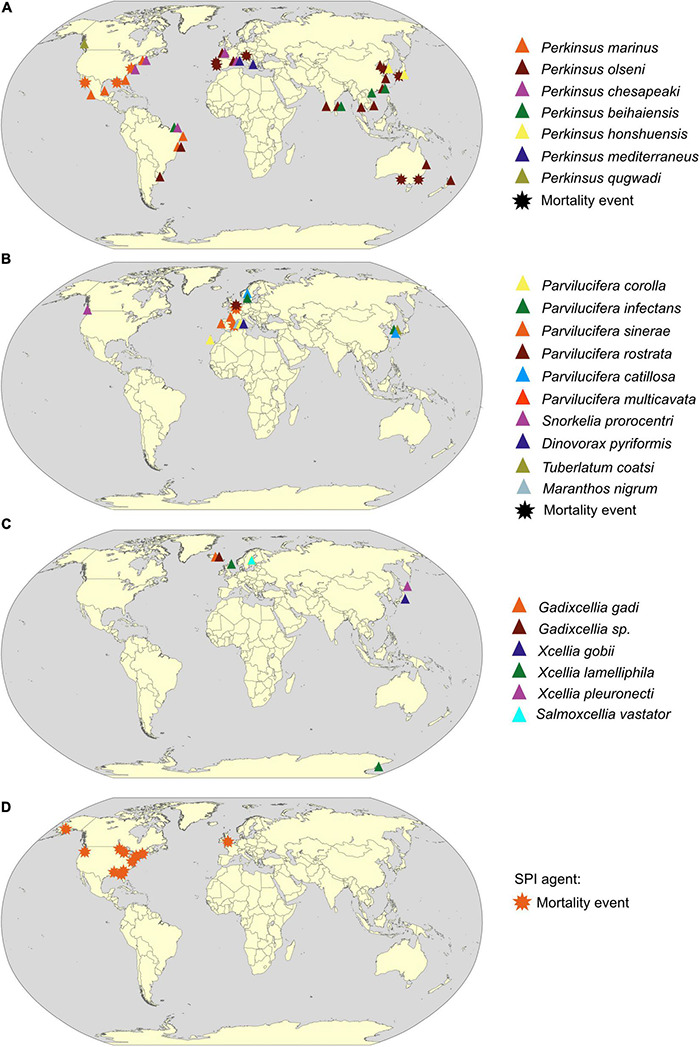
Geographical distribution of Perkinsea parasitic protist based on scientific literature from 1950 to 2020. Detection provenance of **(A)** Perkinsidae, **(B)** *Parvilucifera*ceae, **(C)** Xcellidae family, and **(D)** SPI agent (detail of selected references in Supplementary Table 1) is indicated by a colored triangle when the parasite is detected simultaneously by molecular (qPCR or PCR) and microscopic methodologies (histology or RFTM incubation or cultures), and by a star when presence of the parasitic protists was linked to a mortality event. The color of triangles or stars designates the parasite species (see key). World map drawing is a free public domain vector cliparts (available at https://commons.wikimedia.org).

Until now, most of the host species described above are of commercial interest but other bivalve and gastropod species may also be susceptible to infection, especially in the described geographical range. However, the *Perkinsus* putative wide host range established solely using microscopic and molecular methodology may also be overestimated. Indeed, these results do not allow the identification of dead-end hosts that prevent the parasite transmission to the definite hosts. Furthermore, despite their BHR character, host susceptibility needs to be considered to prevent and protect most sensible bivalve stocks. For example, the two genetically related oysters *C. gigas* and *C. virginica* respond with different susceptibility when challenged by *P. marinus*. Although infection is established in both hosts, only infection in *C. virginica* is lethal (Barber and Mann, 1994). Among *Perkinsus* species, different levels of prevalence depending on hosts species are recorded across the world (Figure 3). In the Chesapeake Bay, *P. marinus* and *P. chesapeaki* infect different species of oysters and clams living in sympatry. Among these hosts, *P. marinus* rarely infects clams compared with *P. chesapeaki* and exhibits oyster preference (Reece et al., 2008). Variability in pathogenicity across the BHR of *Perkinsus* spp. raises the question about the existence of specific strains. For example, such specific strain was recently highlighted for *Perkinsus marinus* with the emergence between 1983 and 1990 of a new hypervirulent phenotypic strain that includes a shortened life cycle and a trophism shift from deeper connective tissues to digestive epithelia (Carnegie et al., 2021). Authors hypothesized that the development of this new strain may be related to reduced oyster abundance and the rapid establishment of the exotic parasite *H. nelsonii* in 1959. Hence, given the increase in epizootic diseases (= disease event in a non-human animal population analogous to an epidemic in humans) worldwide, consideration of the host range is absolutely essential because i) they may be the starting point for a host shift, ii) they represent vector of transmission with no apparent signs of disease, and iii) new native parasitic strains can emerge in response to biotic or abiotic stressors.

**FIGURE 4:**
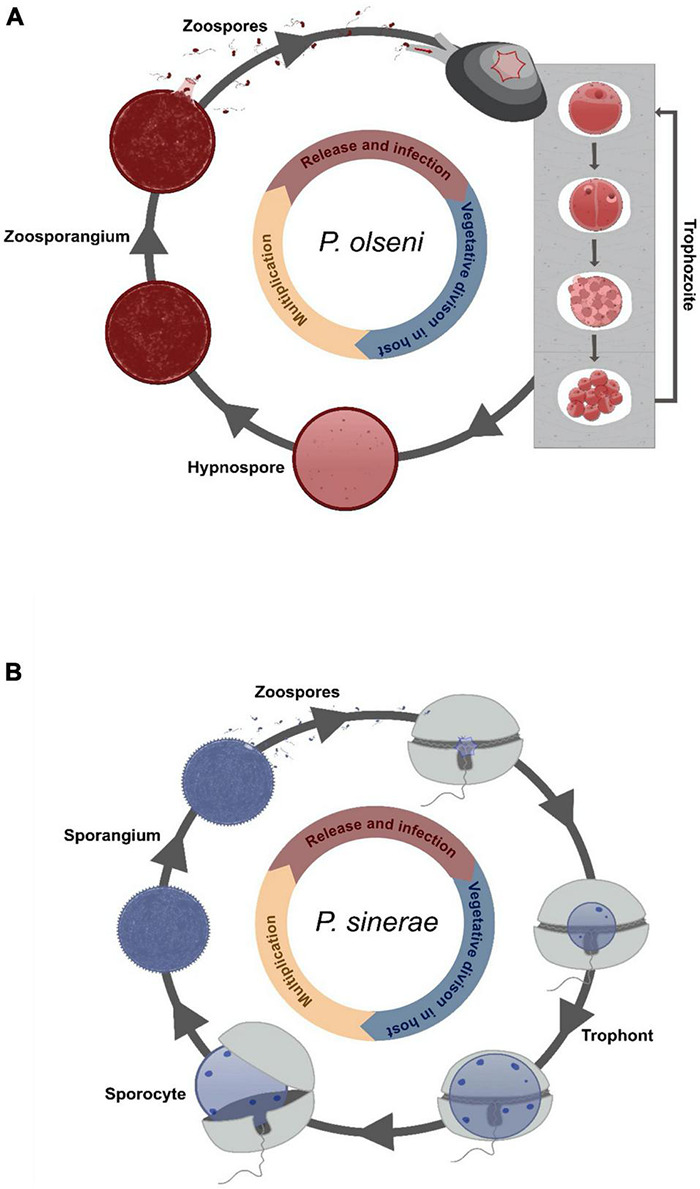
Cartoon illustration of the described life cycle of Perkinsea. Drawing representing the life cycle of **(A)** *Perkinsus olseni* infecting its clam host [adapted from Auzoux-Bordenave et al. (1996)] and of **(B)** *Parvilucifera sinerae* infecting its dinoflagellate host [adapted from Alacid et al. (2015)].

*Perkinsus* infection appears to occur directly by filtration without an intermediate host, with gills and labial palps playing a crucial role as entrance portal for the parasite (Chintala et al., 2002; Allam et al., 2013; Wang et al., 2018). Four different life stages (Figure 4A) are described today occurring inside or outside the host with an exception for *P. qugwadi* where all stages can be observed in host tissues (Blackbourn et al., 1998). All of them can induce new infections into a healthy host. The trophozoite life stage proliferates by vegetative multiplication (palintomy) in the host tissue. Disruption of the cell wall allows the release of immature spherical trophozoites that will gradually enlarge becoming mature vegetative cells (Perkins, 1996). In case of heavy infection, trophozoites can invade totally host tissues, with occasional production of cutaneous white nodules (inflammatory reaction), and induce a global decrease in host fitness (e.g., lower filtration activity, retard in growth and reproduction) (Dittman et al., 2001; Lee et al., 2001; Choi and Park, 2010). When the host becomes moribund, the parasite enlarges and develops a thick cell wall becoming a hypnospore life stage (Valiulis and Mackin, 1969; Perkins, 1996). When transferred to fresh seawater under favorable conditions of temperature and salinity (Auzoux-Bordenave et al., 1996), hypnospores undergo multiple divisions leading to the formation of free-living biflagellate life stages, the zoospores, into a cellular structure called zoosporangium. At maturity, the zoosporangium releases zoospores in the medium allowing the infection of new hosts.

*Perkinsus* species are one of the most famous examples of successful invasive pests in marine environments (Figure 3A). Three different scenarios are described through three different *Perkinsus* species: *(1) Expansion: P. marinus* has shown a significant increase in its geographic area in the United States along the East coast by more than 500 km in 2 years (1990–1992). This expansion, which is now permanent, has been correlated with an increase in winter surface temperature due to milder winters (Ford, 1996; Ford and Smolowitz, 2007). *(2) Introduction: P. chesapeaki* appears to have been accidentally introduced in Europe *via* its vectors *Mya arenaria*, or the hard clam, *Mercenaria mercenaria* from the United States (Arzul et al., 2012). The sporadic detection of *P. chesapeaki* does not reflect an expansion dynamic. To date, no mortality event affiliated to *P. chesapeaki* has been recorded. *(3) Introduction and expansion: P. olseni* has been co-introduced in Europe with its host the Manila clam, *Ruditapes philippinarum*, in 1972 for the development of clam aquaculture (Ruano et al., 2015). After its first detection in the late 1980s, the first mortalities of clam stocks were attributed to this parasite at Ria de Faro in Portugal (Ruano and Cachola, 1986). Since then, *P. olseni* has produced mortalities in Spain, Portugal, and Italy, infecting both the exotic Manila clam, *Ruditapes philippinarum*, and the native European clam, *Ruditapes decussatus* (Azevedo, 1989; Figueras et al., 1992; Pretto et al., 2014). Following these first mortalities, *P. olseni* was detected along the French Atlantic coast with infection prevalence up to 100% (Lassalle et al., 2007), in contrast to the non-detection of *Perkinsus* spp. in most of (these) previously studied sites (Goggin, 1992). Similarly, histological studies conducted by Vilela (1951) on *Minchinia tapetis* (Haplosporidia) infection in the Formosa lagoon in Portugal, where the prevalence of *P. olseni* infection is now very high, ranging from 60 to 90% depending of the season (Leite et al., 2004), did not mention any infection with *Perkinsus* spp. Finally, in 2011, using microsatellite genetic diversity analysis, Vilas et al. described low genetic diversity of the *P. olseni* parasite in Spain and Portugal compared with samples collected in Japan and New Zealand (Vilas et al., 2011), which might result in a founder effect testifying an introduction event of this parasite. These examples of *Perkinsus* spp. dissemination demonstrates the ubiquitous nature (exploitation of an important range of abiotic and biotic niches) of *Perkinsus* species, which is a threat for shellfish farming economy and for ecosystem equilibrium. Bivalves are known to provide important ecosystem services through their status as habitat engineers, as filter feeders, and as a connecting compartment between primary producers and predators (Jones et al., 1996; Coen and Grizzle, 2007; Gallardi, 2014). By cascade effect, parasitic infection can have deleterious effects on the different functions attributed to these populations: (i) by altering their bioturbatory activity and, thus, by modifying biogeochemical cycles at the water–sediment interface (Dairain et al., 2019), (ii) by impacting their filtration activity participating in the nitrogen cycle (= production of NH_4_^+^ leading to an increase in primary production) and helping to reduce turbidity (due to the increase in light penetration) (Gallardi, 2014), and (iii) by altering their physiological traits (e.g., growth, reproduction, and survival), which will modify the structure of habitat, biotic interaction, and the associated species richness (e.g., Thomas and Poulin, 1998). In addition to this infectious threat, benthic biodiversity is threatened by human activities (McCauley et al., 2015). Indeed, anthropogenic eutrophication and temperature increase are all factors that can weaken the immune system of the benthic organisms and lead to a decrease in their resistance to parasitic protists as *Perkinsus* spp. (Morley, 2010). Furthermore, the host range of these parasitic organisms might be wider than described in the scientific literature with the existence of other “non-commercial” potential hosts belonging to suborders infected by *Perkinsus* spp. Thus, even though many arguments allow classifying the *Perkinsus* species as a BHR parasite, we cannot determine the real consequences on stocks of “non-optimal” hosts especially when it is related to sporadic infections with low prevalence. Due to research focus on economically valuable species of mollusks like *C. virginica* or *R. philippinarum*, consequences of these infections on the global host range of *Perkinsus* spp. are still mainly undiscovered.

### Parviluciferaceae, a Group of Parasites of Microalgae

Norén et al. (1999) identified small round structures abnormally present within the toxic dinoflagellate *Dinophysis* spp. on the west coast of Sweden. Molecular and microscopic analyses revealed that this parasitic organism was affiliated to the Perkinsids group and erected a new phylum, the Perkinsea (Norén et al., 1999). So far, five genera have been described and cultivated (Jeon et al., 2018). The first genus described was *Parvilucifera* genus encompassing *P. infectans* (Norén et al., 1999), *P. multicavata* (Jeon et al., 2018), *P. sinerae* (Figueroa et al., 2008), *P. rostrata* (Lepelletier et al., 2014b), *P. corolla* (Reñé et al., 2017b), *P. catillosa*, and *Parvilucifera* sp. (Alacid et al., 2020). Recently, three new genera were described, the genus *Snorkelia* containing *P. prorocentri* (Leander and Hoppenrath, 2008) renamed *Snorkelia prorocentri* (Reñé et al., 2017a), the genus *Dinovorax* including *D. pyriformis* (Reñé et al., 2017a), *Tuberlatum* encompassing *T. coatsi* (Jeon et al., 2018), and finally *Maranthos* including *M. nigrum* (Reñé et al., 2021a). All these genera seem to have a basal position within the Parviluciferaceae group, with the exception of the *Maranthos* species, whose phylogenetic position is not yet clear (Jeon et al., 2018; Reñé et al., 2021a; Figures 1B, 2).

Members of the Parviluciferaceae family share many common traits including development and characteristic of their life cycle, and their infection strategy (Reñé et al., 2017a). The infection begins with the entry of the zoospore into the dinoflagellate host cell (Figure 4B). Once inside, the parasite develops in the cytoplasm forming a spherical cell, the trophont (= trophocyte), which consumes its host for its own growth. At the end of the feeding stage, a free-living non-motile cell, the sporocyte (= immature sporangium), occupying the whole intracellular space, is released by breaking the theca of the host. Next, the cell undergoes morphological transformations into a spherical multinucleated dark structure called the sporangium (= mature sporocyte). The sporangium remains in dormancy until the detection of a host chemical signal (Garcés et al., 2013b). After a short maturation (∼48 h), a sporangium with open aperture(s) releases several hundred new infectious cells (zoospores) (Alacid et al., 2017). To date, all described species are parasites of dinoflagellates including toxic species and possess a BHR (here belonging to a “true” generalist class) (Figueroa et al., 2008; Garcés et al., 2013a; Jeon et al., 2018; Rodríguez and Figueroa, 2020; Reñé et al., 2021b) except *S. prorocentri*, isolated from Boundary Bay (Canada), which was known to only infect the marine benthic dinoflagellate *Prorocentrum fukuyoi* (Leander and Hoppenrath, 2008; Figure 1B). However, in 2017, Reñé et al. isolated a new species of *Snorkelia* from a different location (Catalan Coast, NW Mediterranean) infecting the dinoflagellate *Levanderina fissa* (Reñé et al., 2017a). The host range of *Snorkelia* species, therefore, remains enigmatic. For the “true” generalists, *P. sinerae*, *P. corolla*, and *P. rostrata*, the host range was determined mainly by *in vitro* cross-infection. *In vitro* experiments could result in artificial linkage between a host species and a parasite, which is not representative of the ecological reality (Råberg et al., 2014; Alacid et al., 2016; Reñé et al., 2021b). Infection of non-preferred hosts could be based on chemical and physiological processes shared by several related hosts (e.g., Garcés et al., 2013b). Furthermore, when a parasite infects a host but is not able to produce viable parasitic cells, such “host” cannot be considered as a “true” host for the parasite, e.g., infection of a chlorophyte strain of *Pyramimonas* by *P. corolla* (Rodríguez and Figueroa, 2020). The infection process is probably the result of plesiomorphic mechanisms among *P. corolla*’s hosts, but the parasite cannot achieve its whole life cycle. In this review, we considered *Parvilucifera* species as generalists when they realize a viable life cycle in a large repertoire of host species. A BHR gives them the full potential of host shifting in new environments, although there are evidences of host preference in nature (Alacid et al., 2016). However, much remains to be done to evaluate the contribution of the plesiomorphic and convergent traits of a parasite to the success of generalists.

Despite a “hot-spot” of detection in Europe, the distribution of these parasites of dinoflagellates is worldwide in marine environments (Figure 3B). As major contributors, with diatoms, to the fixation of inorganic carbon through photosynthesis (Falkowski et al., 1998), dinoflagellates are one of the most important components of marine phytoplankton as primary producers and grazers. *Parvilucifera* parasites could participate in regulating dinoflagellate populations in the blooming period, as shown in Spain where 5 to 18% of the population of the noxious dinoflagellates, *Alexandrium minutum*, were killed by *Parvilucifera* sp. during natural bloom (Alacid et al., 2017). For methodological limitation reasons, most studies describing *Parvilucifera* spp. have been carried out when prevalence is the highest on blooming host species, thus, increasing the chances to detect and describe new parasitic species. Unfortunately, this bias hides a possible important part of host and parasite diversity (Reñé et al., 2021b). Indeed, in some studies, the host range of these parasites appears to be much wider, including non-harmful species (e.g., Figueroa et al., 2008; Garcés et al., 2013a; Rodríguez and Figueroa, 2020), even if the *in vitro* specificity tests do not take into account the complexity of planktonic interaction network and the *in situ* parasitic host preferences. This lack of knowledge on *Parvilucifera* distribution, diversity, and trophic niche might become problematic, given its BHR nature and the global trades. Indeed, ballast waters, which are one of the most important vectors of introduction in marine systems (Carlton, 1985; Barry et al., 2008), could contribute to the dispersal of *Parvilucifera* trophonts or dormant sporangia (e.g., Darling et al., 2018), thus, increasing the risk of settlement in new areas. Hence, introductions of these BHR parasites could act positively as a top–down control of the toxic dinoflagellate blooming species and, conversely, could play a key role in deregulating the planktonic compartment given the importance of dinoflagellates as primary producers inducing significant shifts in marine food webs.

### Xcellidae, a New Group of Fish Parasites

In 1969, Brooks et al. of two flatfish species, the black-tipped flounder, *Psettichthys melanostictus*, and the Pacific plaice, *Platichthys stellatus* (Brooks et al., 1969). Thirty-five years later, a protozoan named X-cell was identified as responsible for this pathology inducing also multiple lesions and swelling of the gill filaments (Miwa et al., 2004). Using small and large concatenated subunit (SSU and LSU) rDNA phylogeny reconstruction, Freeman et al. (2017) investigated the phylogenetic position of this enigmatic X-cell protist and showed that it formed two highly distinct clades, one corresponding to the pseudobranchial parasites of Gadiformes, and the other to gill and epidermal X-cells from Perciforms and Pleuronectiformes called *Gadixcellia* and *Xcellia*, respectively. More recently, Karlsbakk et al. (2021) described a new genus named *Salmoxcellia*, a sister group to *Gadixcellia*. Despite an unusually high genetic divergence between the three genera, with a similarity of 74.9% for the SSU rDNA sequences, these two close sister clades form a new family called Xcellidae (or Xcellins) branching within the Perkinsea lineage (Freeman et al., 2017; Karlsbakk et al., 2021) (Figure 2B).

Histological examination and scanning electron microscopy of xenomas of infected fish revealed a large number of clustered round parasitic cells surrounded by host connective tissue. Currently, only one developmental life stage has been described up to today; hence, the life cycle of this parasite still remains unknown (Freeman et al., 2017). However, Freeman et al. (2017) suggested that infection occurs *via* contact between fish and the benthos. Indeed, X-cell infection occurs in fish species with at least one benthic stage during their life cycle (Fahay, 1983; Lough et al., 1989). An experimental infection *in situ* in tanks revealed that only fish exposed, even transiently, to the benthos were infected and showed symptoms of disease (Eydal et al., 2010). The infected fish samples are mainly young and adult individuals. Indeed, the first pathogenic symptoms (pseudobranchial xenomas) appear macroscopically in young wild cod (around 6 months old, 6- to 13-cm length) from Icelandic waters with a prevalence peak of 23% at the age of 22 months, whereas older or larger fish (40- to 76-cm length) have a lower prevalence around 7 and 1%, respectively (Eydal et al., 2010). Similar trends had been observed in Atlantic cod (Morrison et al., 1982) and the Pacific cod (Stich et al., 1976). However, the question remains open whether the mortality induced by the X-cell infectious agent on juveniles could remove an age class from the field sampling and bias the observed prevalence. Although mortality induced by these infectious agents still needs to be demonstrated, the transfer of wild infected individuals of *Gadus morhua* in tanks has shown an important mortality (Eydal et al., 2010).

Parasites belonging to these three genera have been detected in more than 20 fish species belonging to five orders of teleost: Pleuronectiformes (flatfishes) (Freeman, 2009; Freeman et al., 2011), Perciformes (perch-like fish) (Katsura et al., 1984), Gadiformes (cods) (Freeman et al., 2011), Siluriformes (catfishes) (Diamant et al., 1994), and Salmoniformes (salmonids) (Dyková et al., 1993; Karlsbakk et al., 2021). Until now, five species have been described (Figure 1B). The parasite *Xcellia pleuronecti* infection has been confirmed in *Hippoglossoides dubius* and *Pseudopleuronectes obscurus* but also suspected in many other species from the same area: *Cleisthenes herzensteini*, *C. pinetorum*, *Glyptocephalus stelleri*, *Kareius bicoloratus*, *Hippoglossoides elassodon*, *Liopsetta pinnifasciata*, *Platichthys stellatus*, *Parophrys vetulus*, *Pseudopleuronectes schrenki*, and *Verasper moseri*. Equally, *Xcellia lamelliphila* infection is detected in *Limanda limanda*, *Lycodes* spp., *Macruronus novaezelandiae*, *Merluccius gayi gayi*, and *Trematomus* spp. (Freeman et al., 2017). Finally, the newly described parasite *Salmoxcellia vastator* is detected in *Oncorhynchus mykiss* and in *Salmo salar* (Karlsbakk et al., 2021). These three parasites are, therefore, BHR parasites (Figure 1B). Conversely, *Xcellia gobii* and *Gadixcellia gadi* infecting, respectively, *Acanthogobius flavimanus* and *Gadus morhua* are NHR parasites and classified as specialists (Freeman et al., 2017; Figure 1B). This classification will certainly evolve as new knowledge on Xcellidae group becomes available.

In addition, Xcellidae protists have been detected in a restricted geographical area (Northern Europe and Japan) (Figure 3C), but the monitoring of these X-cell infections is hard to carry out and stay largely incomplete because of migratory fish hosts in the worldwide ocean. Their host range is mainly unknown, but we can hypothesize that it may be broader than that currently identified. An extended host range would not be surprising considering the Perkinsea phylum constituted of a majority of BHR parasites closely related phylogenetically (e.g., the Perkinsidae) (Figure 2B). Overall, the consequences of the X-cell infection on fish populations are not well known. However, as it affects important halieutic resources as described for the salmonid parasite, *Salmoxcellia* vastator, it makes farmed salmonid fillets unsuitable for sale (Karlsbakk et al., 2021). Moreover, these parasites could infect vulnerable species, such as Atlantic cod fish *G. morhua*, extensively farmed, but mainly in the north of Europe (see FAO, 2021 Programme—*Gadus morhua*). Commercially important fish, a concern of the global trades, which could act as reservoirs allowing the dissemination in farmed and wild fish populations.

### Severe Perkinsea Infection, an Infectious Agent of Tadpoles Populations

With up to 50% of all threatened species, amphibian populations are emblematic representatives of the sixth mass extinction event to which highly virulent wildlife diseases contribute (Stuart et al., 2004; Gewin, 2008; Chambouvet et al., 2020). Recent works have mainly identified two pathogens as drivers of this decline, the fungus *Batrachochytrium dendrobatidis* and the Ranavirus (e.g., Bosch et al., 2001; Fisher et al., 2009; Gray et al., 2009; Olson et al., 2013).

However, in April 2006, in a pond in northeast Georgia (United States), Davis et al. (2007) observed a massive mortality event of southern leopard tadpoles, *Rana sphenocephala* (syn. *Lithobates sphenocephalus*), attributed to an unknown protist affiliated by phylogenetic analysis to the Perkinsea lineage. Using phylogenetic analysis, it has been shown that this organism belongs to a discrete clade named pathogenic Perkinsea clade (PPC) (Isidoro-Ayza et al., 2017) within the wider monophylic cluster of the Novel Alveolate Group 01 (NAG01) (Chambouvet et al., 2015) obtained from disparate freshwater environments or internal organs (e.g., liver tissues) of a wide variety of Neobatrachia suborder (Chambouvet et al., 2015; Isidoro-Ayza et al., 2017; Figures 1B, 2B). Infectious agents of the PPC clade have been identified as responsible for the die-offs of tadpole throughout the United States (Isidoro-Ayza et al., 2017). Infected tadpoles with pathological symptoms, named “severe Perkinsea infection” (SPI), showed lethargic swimming with enlarged and histopathologic lesions of the liver, mesonephros, spleen, pancreas, gills, gastrointestinal tract, skeletal muscle, dermis, and peritoneum (Green et al., 2002; Davis et al., 2007; Jones et al., 2012; Isidoro-Ayza et al., 2017). Histological examination of infected liver tissues revealed a massive number of round infective cells replacing the normal hepatic parenchyma. Two distinct putative life stages were identified invading the hepatocyte cells corresponding to hypnospore-like and trophozoite-like life stages (Isidoro-Ayza et al., 2017, 2019). SPI symptoms have been reported from summer to early autumn in boreal and temperate regions, and from late winter to early spring in subtropical areas (Isidoro-Ayza et al., 2017; Figure 3D). Although this parasite is mainly described as infecting tadpole life stage, one report highlights infection of the adult populations with granulomatous lesion in the legs (Jones et al., 2012). These results suggest that either most of the tadpoles die before the metamorphosis or that mature immune systems acquired after the metamorphosis may drive back the infection (Isidoro-Ayza et al., 2017). Although the relationship between the infectious agent and the disease is not yet well established, symptoms of the disease could result from co-infection between the Perkinsea parasite and others infectious agents, such as the FV3-like virus, responsible for frog population declines, which infects both larval and adult amphibians (Gray et al., 2009; Lesbarrères et al., 2012), and/or alteration of tadpole immune systems (Isidoro-Ayza et al., 2017).

During monitored SPI outbreaks, mortality rate can reach up to 95% of the population leading to the loss of an entire age class or, in case of chronic infection, to a reduced recruitment (Green et al., 2002; Isidoro-Ayza et al., 2017, 2019). Now recognized as the third most common infectious disease of anuran species, SPI infectious agent is described as an emerging pathogen. It is therefore now fundamental to understand the relationship between this infectious agent and the outcome of the disease and how other pathogen communities and/or environmental factors could affect disease susceptibility. Furthermore, as the globalization of trade where amphibian species, generally involved in meat or pet trade, could spread the parasitic invaders *via* reservoir species into native and/or naive amphibian populations, the study of this pathogen is crucial.

### Environmental Diversity: Unveiling Putative Pathogens?

Molecular methodologies have recently highlighted that our vision of Perkinsea diversity, mainly assessed by culture-based methods, is still only the tip of the iceberg. In both marine and freshwater environments, analysis of genetic diversity based on SSU rDNA sequences of the smallest sizes of plankton (<5 μm), revealed a previously unknown diversity of these organisms (Moon-van der Staay et al., 2001; López-García et al., 2001, 2003; Lefranc et al., 2005; Mangot et al., 2009).

A recent overview of freshwater Perkinsea genetic diversity, especially in lakes, revealed that this group appears to be diverse and abundant, with an observation of 2,927 operational taxonomic units (OTUs) defined at 95% of sequence similarity, which represented 3% of the eukaryotic diversity analyzed in Debroas et al. (2017), suggesting an important role in the trophic web (e.g., Lepère et al., 2008, 2011; Jobard et al., 2020). Recent studies have highlighted that freshwater clades could be a parasitic protist of the colonial green algae *Sphaerocystis* sp. (Jobard et al., 2020). These results are consistent with the description of *Rastrimonas* gen. nov. (Brugerolle, 2003), previously described as *Cryptophagus subtilis*, infecting the free-living Cryptophyte *Chilomonas paramaecium* (Brugerolle, 2002). However, apart from these two descriptive publications, no molecular analysis has yet been carried out to definitively affiliate *Rastrimonas* sp. within the Perkinsea lineage.

In marine environments, molecular signatures of Perkinsea were also found in extreme environments, such as hydrothermal vents or anoxic fjords (López-García et al., 2003; Zuendorf et al., 2006), but most of environmental genomics studies target water column samples [e.g., surface or deep chlorophyll maximum (DCM) depths] (e.g., Moon-van der Staay et al., 2001; López-García et al., 2003; De Vargas et al., 2015). This lack of genetic signatures in marine environmental surveys represent a real paradox, since the two main cultivable groups described, *Perkinsus* spp. and *Parvilucifera* spp., are marine. In 2014, targeting the V4 hypervariable region of the rDNA and rRNA templates using 454 sequencing technology, Chambouvet et al. (2014) evaluated the genetic diversity of the eukaryotic microbial community in two sampling depths (surface and DCM) and in the sediment across four different locations across Europe (Oslo, Norway; Naples, Italy; Barcelona, Spain; Roscoff and France). The analysis revealed an unexpected genetic diversity of ribosomally active organisms belonging to Perkinsea other than than *Parviluciferaceae* and *Perkinsus* clusters mainly detected in the sediment with a total of 265 sequences clustered in 150 OTUs defined at 99% similarity. The ribosomal RNA sequences present in these clades belong to metabolically active organisms, which certainly play an active role in the ecosystem functioning. This cryptic diversity, only detected by their genetic signatures, raises new scientific questions about the putative existence of non-parasitic heterotrophic organisms, the host range of parasitic Perkinsea, and their role and impact on the aquatic food web. Finally, these results suggested that sediments can act as parasite reservoirs, as suggested for *Amoebophrya* parasitoids (Chambouvet et al., 2011) and for marine Perkinsea (Chambouvet et al., 2014; Freeman et al., 2017; Reñé et al., 2021b). However, studies on diversity and distribution are scarce and restricted to specific environments (e.g., coastal water or few freshwater environments). A wider study of the Perkinsea distribution could give a more complete picture of environmental distribution unveiling potential reservoirs of pathogens. A phylogenetic analysis of Perkinsea illustrates two main evolutionary divisions between clades associated to Perkinsidae and Xcellidae, and Parviluciferaceae prior to marine/freshwater specialization (Jobard et al., 2020). This also suggests that transitions between marine and freshwater were few in the life history of Perkinsea, as differences between these environments constitute a barrier for cross-colonization for most organisms (Logares et al., 2009; Bråte et al., 2010). However, a deep analysis of environmental DNA may reveal new information on the evolution and environmental colonization of Perkinsea.

## Conclusion

All currently known members of Perkinsea are described as parasitic species infecting protists, mollusks and vertebrates. They are detected in all ecosystems from the tropics to high latitudes and from freshwater to marine environments (Bråte et al., 2010; Mangot et al., 2011). Perkinsea share common characteristics, which represent serious threats for biodiversity and human activity.

(1) **They are putatively predominantly BHR.** Perkinsea consist of four described lineages of putative BHR parasites, with strong evidences of host preference (e.g., Garcés et al., 2013a) or susceptibility (e.g., Calvo et al., 1999). At high taxonomic level, such homogeneity is rare compared with other major parasitic clades in ecosystems. Indeed, some strains of *Amoebophrya* species (Syndiniales, Alveolata) exhibit different specificity to host species leading to a mix of parasitic protist displaying various degrees of host specialization (Coats and Park, 2002; Chambouvet et al., 2008; Farhat et al., 2018). In the same way, while many chytrid fungi (Chytridiomycota) infecting phytoplankton are mainly considered as host specific (Ibelings et al., 2004), *Dinomyces arenysensis* has a BHR focused on dinoflagellate species (Lepelletier et al., 2014a). The same trend is observed for Haplosporidae, which is one of the major pests involved in mollusk diseases with the Perkinsidae, which is composed of NHR and BHR. For example, *Bonamia* species infects several oyster species (Engelsma et al., 2014) whereas *Haplosporidium* species are able to infect distant phylogenetic hosts, including mollusks, crustaceans, or even polychaetes (Arzul and Carnegie, 2015). This peculiar putative BHR dominant characteristic is probably strongly related to their common evolutionary history and may represent an ecological advantage that enable survival improvement *via* a wide variety of “potentials” hosts. However, this assumption should be taken into caution as variability in pathogenicity could also rely on the existence of specific strains within a species. For example, *Marteilia refringens* (Paramyxin), responsible of marteiliosis, exhibits a potential profile of BHR parasite because this microeukaryotic parasite infects oysters, mussels, and clams. Nonetheless, three different strains have been observed: the “O” strain preferentially found in oysters, the “M” strain mainly found in mussels, and finally the recent “C” strain infecting the cockles *Cerastoderma edule* (Le Roux et al., 2001; Carrasco et al., 2012; Arzul et al., 2014; Guo and Ford, 2016). Hence, the putative BHR of Perkinsidae, Parviluciferaceae, and SPI agents may finally represent the tip of the iceberg with a more complex reality that needs to be urgently explored because of consequences in terms of the development and implementation of new strategies in disease and conservation management.
(2) **They are pathogenic for many keystone, engineer, or endangered species.** Like other important parasites listed at international or national level (e.g., *Batrachochytrium dendrobatidis* or *Marteilia refringens*), Perkinsea clearly contributes to the loss and dismiss of some species (e.g., the SPI agent) and to the massive loss of fishery and aquaculture resources (e.g., *P. olseni* and *P. marinus*). Recent years have seen the advent of diseases induced by *Batrachochytrium dendrobatidis* and *B. salamandrivorans* (agent of chytridiomycosis), listed in the OIE-notifiable disease list, which are mainly responsible for the collapse of amphibian populations (Scheele et al., 2019). In 2020, Chambouvet et al. reviewed new infectious diseases concerning Apicomplexans (Coccidians, Gregarines) and Perkinsea (SPI agent) that may worsen amphibian situation. Today, many species of frogs, such as *Lithobates capito* or *L. sevosus*, have been listed as “Near threatened” to “Critically endangered” status on the International Union for Conservation of Nature (IUCN) red list (Chambouvet et al., 2020). Their involvement in this loss may contribute to major shifts in biological communities.
(3) **They are responsible for economical loss.** Economic valuable species are affected by Perkinsea infection, which may lead to collapse of fishery and aquaculture industries. As protozoans caused most of the historically significant diseases in molluscs, this parasitic group is of major concern for the shellfish exploitation (Carnegie et al., 2016). The Dermo disease (etiological agent: *P. marinus*) is one of the major marine molluscan diseases in addition to MSX (multinucleated sphere unknown caused by *Haplosporidium nelsoni*), marteiliosis (*Marteilia refringens* and *M. sydneyi*), and bonamiosis (*Bonamia ostreae* and *B. exitiosa*) (Arzul and Carnegie, 2015; Guo and Ford, 2016). Globally, Haplosporidia and Perkinsidae are two major threats for mollusk health even if the mortality rate produced by *P. marinus* (max. 60%) on oysters is lower than the mortality rate of *Haplosporidium nelsoni* or *Marteilia refringens* (90–100%) on the same resource (Guo and Ford, 2016). Indeed, in the Chesapeake Bay, the oyster harvest decline (∼80.000t between 1910 and 1980 to 15.000t in 1986) was attributed to both parasites *P. marinus* and *H. nelsoni* in a context of inadequate management practices (Héral et al., 1990; Goulletquer et al., 1994). Recurrent declines in clam harvests are recorded as a result of massive mortalities caused by *P. olseni* in Korea (Park et al., 1999; Choi and Park, 2005), China (YuBo et al., 2001), and Japan (Hamaguchi et al., 1998). The Manila clam landings from culture in 1997 was approximately 14.000t, which is only one-fifth of the clam landings in 1990 (Park and Choi, 2001). In Europe (Spain, Portugal, and Italy), the parasite destroyed *R. decussatus* (native) and *R. philippinarum* (exotic) populations (Azevedo, 1989; Figueras et al., 1992; Pretto et al., 2014). Perkinsosis induces more damage to clam stocks than other important diseases, such as the brown ring disease (BRD), producing approximately 20% of mortality over 2 years (Guo and Ford, 2016).
(4) **They are easily translocated and could be invasive.** Today, many precautions (e.g., quarantine of animals) must be taken around the world to stem the spread of Perkinsea, particularly with regard to Perkinsidae. Since the first mortalities, *P. marinus* has been listed as a notifiable pathogen by the OIE (Oie-Listed Diseases, 2021: OIE—World Organisation for Animal Health) and the European Commission (Directive 2006/088/EC). However, *P. olseni* is solely classified at the international level in the OIE—list of notifiable diseases but is out of concern for the European Commission (Carnegie et al., 2016). Some European countries are *Perkinsus olseni* free, and its exclusion from the notifiable disease list from the European Commission can lead to relaxed vigilance within the trading network contributing to its spread in European non-affected areas (Carnegie et al., 2016). National surveillance efforts are different between European member states, and mortality events are mainly reported by shellfish farmers. However, these networks have already revealed pathogens in new areas, like OsHV-1 μvar or *B. ostreae*, or new pathogens implied in shellfish mortalities, like *Mikrocytos* species. Therefore, given the putative wide host range of *P. olseni* and other Perkinsea species, the detection of new pathogens in new areas should be monitored using next-generation sequencing methodologies with confirmation by classical microscopic techniques.
(5) **Most Perkinsea are only described by their genetic signatures**.
  Environmental sequencing revealed a high diversity of several potential microeukaryotic parasites in the water column and sediments (Chambouvet et al., 2014). These results show that the study of environmental diversity is absolutely crucial to identify the potential distribution and emergence of parasites. Thanks to molecular methods, some of problematical parasitic groups, e.g., the haplosporidians, are under survey (Hartikainen et al., 2014; Bass et al., 2015). Recently, *Haplosporidium diporeiae* infecting amphipods was associated to the previously described environmental clade “haplosporidian clade C” (Hartikainen et al., 2014; Winters and Faisal, 2014; Bass et al., 2015). It is now clear that Perkinsea lineage is genetically diverse in aquatic environments and may be composed of clades with a pathogenic potential whose hosts have not yet been identified. This environmental diversity described across different clusters (e.g., Chambouvet et al., 2014) clearly represents potential parasites. However, their host range and impact of the aquatic food webs are still black boxes that the scientific community need to urgently address considering the impact of already described BHR species belonging to this lineage.

In a context of intensification of the global trade, this opportunistic BHR parasitic protists represent a threat to become successful invasive species eventually leading to a new putative emerging disease. It is thereby now important to investigate their full host range, their cryptic diversity, and their role in the global aquatic network. On the other hand, a growing body of evidence emphasizes the importance of the interactive network between a host, the whole associated microbial communities (including others parasites), and the environmental conditions in the determination of infection outcome. Indeed, the outdated “one parasite, one disease” paradigm is not sufficient to explain a disease, and therefore, these parasites and their studies should be integrated into a larger scheme (Bass et al., 2019). Knowing under what conditions invasion by these infectious agents could be successful in and what threats they could pose to native host populations are two more fundamental questions that need to be addressed.

## Author Contributions

SI, PS, and AC designed the study. SM performed the phylogenetic analyses. AC provided the funds. All authors drafted the manuscript and approved the final version of the manuscript.

## Conflict of Interest

The authors declare that the research was conducted in the absence of any commercial or financial relationships that could be construed as a potential conflict of interest.

## Publisher’s Note

All claims expressed in this article are solely those of the authors and do not necessarily represent those of their affiliated organizations, or those of the publisher, the editors and the reviewers. Any product that may be evaluated in this article, or claim that may be made by its manufacturer, is not guaranteed or endorsed by the publisher.
