# Supplementary material for: Emerging Parasitic Protists: The Case of Perkinsea
Source: Front Microbiol. 2022 Jan 13;12:735815. doi: 10.3389/fmicb.2021.735815 (PMC8792838; doi:10.3389/fmicb.2021.735815)
Supplement: Supplementary file 1 [file Data_Sheet_1.docx]

**Emerging parasitic protists: the case of Perkinsea**

Sarah Itoïz^1^, Sebastian Metz^1^, Evelyne Derelle^1^, Albert Reñé^2^, Esther Garcés^2^, David Bass^3, 4, 5^, Philippe Soudant*^1^& Aurélie Chambouvet*^1, 6^

**SUPPORTING MATERIALS AND METHODS**

**Multiple sequence alignment and phylogenetic analysis of the Perkinsea SSU sequences.**

The Perkinsea SSU rDNA phylogenetic tree was performed with published sequences from 134 taxa including 127 cultured and environmental sequences related to Perkinsea and 7 to dinoflagellates as outgroups (AF022200.1, AB264776.1, AF080096.1, M88521.1, U27500.1, AF472555.1, EF065717.1). The sequences were aligned using Q-INS-i interactive refinement method implemented in MAFFT v7.475 (Katoh and Standley, 2013). The alignment was then checked and masked using SeaView (Gouy et al., 2010), resulting in a data matrix of 134 sequences and 1431 alignment positions. The phylogenetic analysis was performed with IQ-Tree v2.0.3 (Minh et al., 2020). The best model was selected by ModelFinder (Kalyaanamoorthy et al., 2017), implemented in IQ-Tree (-MFP option). The selected model was GTR+F+R5. The Maximum-Likelihood phylogeny was estimated using the SH-like approximate likelihood ratio test (Guindon et al., 2010) and 1,000 bootstrap values for branch support (-alrt 1000 parameter).

Bayesian posterior probabilities were calculated using MrBayes v3.2.6 (Huelsenbeck and Ronquist, 2001) on the CIPRES science gateway (available at http://www.phylo.org/). The model GTR + Γ + R5 (lset, nst=6 rates=gamma,ngammacat=5) was selected. The chains were run for 8,000,000 generations with two replicate tree searches both with 4 MCMC chains with a heat parameter of 2. Trees were sampled every 200 generations. In both analyses, the first 25% of the generations sampled were discarded (as the burnin). The consensus topologies and posterior probabilities of each node were then calculated from the remaining trees.

The annotation of the ML tree was performed using Interactive Tree of Life (IToL) (https://itol.embl.de/, Letunic and Bork, 2019) and Inkscape (<https://inkscape.org/en/>). For each branch, support values were notated using the following convention: support values are summarized by black circles when ≥ 80%/0.9 and white circles when it is not the case but values ≥60%/0.6. When the topology is inconsistent in one of the inference methods, it is denoted by a “-”. The sequence origins are represented by squares of colors: light blue for marine waters, yellow for land waters, green for brackish waters and red for wetland soil. Purple asterisks indicate host associated sequences. The alignment and the estimated trees are available in Github (https://github.com/sebametz/Perkinsea-Tree).

**References:**

Capella-Gutierrez, S., Silla-Martinez, J. M., and Gabaldon, T. (2009). trimAl: a tool for automated alignment trimming in large-scale phylogenetic analyses. *Bioinformatics* 25, 1972–1973. doi:10.1093/bioinformatics/btp348.

Gouy, M., Guindon, S., & Gascuel, O. (2010). SeaView version 4: a multiplatform graphical user interface for sequence alignment and phylogenetic tree building. *Mol. Biol. Evol.*, 27, 221-224. doi:10.1093/molbev/msp259

Guindon, S., Dufayard, J.-F., Lefort, V., Anisimova, M., Hordijk, W., and Gascuel, O. (2010). New algorithms and methods to estimate maximum-likelihood phylogenies: Assessing the performance of PhyML 3.0. *Syst. Biol.* 59, 307–321. doi:10.1093/sysbio/syq010.

Huelsenbeck, J. P., and Ronquist, F. (2001). MRBAYES: Bayesian inference of phylogenetic trees. *Bioinformatics* 17, 754–755. doi:10.1093/bioinformatics/17.8.754.

Kalyaanamoorthy, S., Minh, B. Q., Wong, T. K. F., von Haeseler, A., and Jermiin, L. S. (2017). ModelFinder: fast model selection for accurate phylogenetic estimates. *Nat. Methods* 14, 587–589. doi:10.1038/nmeth.4285.

Katoh, K., and Standley, D. M. (2013). MAFFT Multiple sequence alignment software version 7: improvements in performance and usability. *Mol. Biol. Evol.* 30, 772–780. doi:10.1093/molbev/mst010.

Letunic, I., and Bork, P. (2019). Interactive Tree Of Life (iTOL) v4: recent updates and new developments. Nucleic Acids Res. 47, W256–W259. doi:10.1093/nar/gkz239.

Minh, B. Q., Schmidt, H. A., Chernomor, O., Schrempf, D., Woodhams, M. D., von Haeseler, A., et al. (2020). IQ-TREE 2: New models and efficient methods for phylogenetic inference in the genomic era. *Mol. Ecol. Evol.* 37, 1530–1534. doi:10.1093/molbev/msaa015.
