# Supplementary material for: Emerging Parasitic Protists: The Case of Perkinsea
Source: Front Microbiol. 2022 Jan 13;12:735815. doi: 10.3389/fmicb.2021.735815 (PMC8792838; doi:10.3389/fmicb.2021.735815)
Supplement: Supplementary file 2 [file Data_Sheet_2.docx]

Supplementary Material

**Supplementary Table S1.** Host range and sources of occurrences and of Perkinsidae, Parviluciferaceae, SPI agent and Xcellidae detected by both molecular (qPCR or PCR) and microscopical (histology or RFTM incubation or cultures) methodologies.

| **Clade** | **Species** | **Host** | **Continent** | **Country** | **Location** | **Reference** |
| --- | --- | --- | --- | --- | --- | --- |
| Perkinsidae | *Perkinsus olseni* | *Ruditapes decussatus, R. philippinarum, Venus verrucosa* | Europe | Spain, France, Portuga, Italia | Mediterranean Sea, Atlantic Ocean | (Azevedo, 1989; Figueras et al., 1992; Elandaloussi et al., 2009; Arzul et al., 2012; Pretto et al., 2014; Ramilo et al., 2015) |
| Perkinsidae | *Perkinsus olseni* | *R. decussatus* | Africa | Tunisia | Southern Mediterranean Sea | (El Bour et al., 2012) |
| Perkinsidae | *Perkinsus olseni* | *R. philippinarum, Protothaca jedoensis, Tridacna crocea, Pictada fucata, Paphia malabarica* | Asia | China, Korea, India, Japan, Vietnam | Yellow Sea, Bohai Sea, East China Sea, Sea of Japan, Bay of Bengal, Lakshadweep Sea, Pacific Ocean | (Hamaguchi et al., 1998; Park et al., 2006, 2008; Sheppard and Phillips, 2008; Choi and Park, 2010; Sanil et al., 2010; Wu et al., 2011; Shamal, 2018) |
| Perkinsidae | *Perkinsus olseni* | *Pitar rostrata, Crassostrea rizophorae, C. gasar* | South America | Brazil, Uruguay, Panama | Atlantic Ocean, Caribbean Basin | (Cremonte et al., 2005; Sabry et al., 2009; da Silva et al., 2014) |
| Perkinsidae | *Perkinsus olseni* | *Haliotis laevigata, H. rubra, Austrovenus stutchbutyi* | Oceania | Australia, New-Zealand | Indian Ocean, the Coral Sea, Pacific Ocean | (Goggin and Lester, 1995; Murrell et al., 2002; Dungan et al., 2007) |
| Perkinsidae | *Perkinsus marinus* | *C. virginica, C. gigas, Saccostrea palmula* | North America | USA, Mexico | Chesapeake Bay, Gulf of Mexico, Florida coast Delaware bay, Gulf of California | (Marsh et al., 1995; Reece et al., 2001; Gullian-Klanian et al., 2008; Enríquez-Espinoza et al., 2010; Cáceres-Martínez et al., 2012) |
| Perkinsidae | *Perkinsus marinus* | *C. gasar, C. rhizophorae* | South America | Brazil | Atlantic Ocean | (da Silva et al., 2013, 2014) |
| Perkinsidae | *Perkinsus chesapeaki* | *Mya arenaria, Macoma balthica, Tagelus plebeius, C. virginica, Cyrtopleura costata* | North America | USA | Chesapeake Bay, Gulf of Maine | (Mclaughlin et al., 2000; Coss et al., 2001; Reece et al., 2008; Marquis et al., 2020) |
| Perkinsidae | *Perkinsus chesapeaki* | *C. rhizophorae* | South America | Brazil | Atlantic Ocean | (Dantas Neto et al., 2016) |
| Perkinsidae | *Perkinsus chesapeaki* | *R. decussatus, R. phillipinarum* | Europe | France | Mediterranean Sea, Atlantic Ocean | (Arzul et al., 2012) |
| Perkinsidae | *Perkinsus beihaiensis* | *C. hongkongensis, C. ariakensis, C. madrasensis* | Asia | China, India | South China Sea, Bay of Bengal | (Moss et al., 2008; Sanil et al., 2012) |
| Perkinsidae | *Perkinsus beihaiensis* | *Anomalocardia brasiliana* | South America | Brazil | Atlantic Ocean | (Pinho Ferreira et al., 2015) |
| Perkinsidae | *Perkinsus honshuensis* | *R. phillipinarum, R. variegatus* | Asia | Japan, Korea | Pacific Ocean | (Dungan and Reece, 2006; Kang et al., 2016) |
| Perkinsidae | *Perkinsus mediterraneus* | *Ostrea edulis, Arca noae, V. verrucosa, chlamys varia* | Europe | Spain, Italy | Mediterranean Sea | (Casas et al., 2004; Ramilo et al., 2015) |
| Perkinsidae | *Perkinsus qugwadi* | *Patinopecten yessoensis* | North America | Canada | Gulf of Alaska | (Itoh et al., 2013) |
| Parviluciferaceae | *Parvilucifera sinerae* | *Gymnodinium catenatum, G. nolleri, G. aureolum, Karenia brevis, Alexandrium andersonii, A. catenella, A. insuetum, A. margalefii, A. minutum, A. tamutum, A. ostenfeldii, A. peruvianum, Protoceratium reticulatum, Gonyaulax spinifera, Scrippsiella donghaienis, S. trochoidea, Prorocentrum micans, Akashiwo sanguinea* | Europe | Spain, France, Portugal | Mediterranean Sea, Atlantic Ocean, English Channel | (Figueroa et al., 2008; Lepelletier et al., 2014; Garcés and Hoppenrath, 12010) |
| Parviluciferaceae | *Parvilucifera rostrata* | *Alexandrium minutum, A. catenella, A. ostenfeldii, A. tamutum, Gonyaulax spinifera, Gymnodinium aureolum, Heterocapsa triquetra, Scrippsiella trochoidea, Akashiwo sanguinea, Prorocentrum micans* | Europe | France | English Channel | (Lepelletier et al., 2014) |
| Parviluciferaceae | *Parvilucifera corolla* | *Durinskia baltica, Akashiwo sanguinea, Gymnodinium catenatum, G. impudicum, G. microreticulatum, G. nolleri, Heterocapsa pygmaea, Lepidodinium chlorophorum, Levanderina fissa, Lingulodinium polyedrum, Ostreopsis cf. ovata, O. fattorussoi, O. lenticularis, A. andersonii, A. catenella, A. minutum, A. andersoni, A. mediterraneum, A. tamutum, Amphidinium carterae, Barrufeta bravensis, Coolia canariensis, C. monotis, C. palmyrensis, C. tropicalis, Dinophysis acuminata,Prorocentrum micans, P. triestinum, Protodinium simplex, Scrippsiella acuminata, Takayama sp.* | Africa | Spain | Canary Islands, Mediterranean Sea | (Reñé et al., 2017b; Rodríguez and Figueroa, 2020) |
| Parviluciferaceae | *Parvilucifera catillosa* | *Kryptoperidinium foliaceum, Heterocapsa triquetra* | Europe | Sweden | Baltic Sea | (Alacid et al., 2020) |
| Parviluciferaceae | *Parvilucifera infectans* | *Dinophysis acuminata, Alexandrium fundyense, A. tamarense, A. ostenfeldii,* | Europe | Sweden | North Sea, Baltic Sea | (Norén et al., 1999; Johansson et al., 2006) |
| Parviluciferaceae | *Parvilucifera infectans* | *Akashiwo sanguinea* | Asia | Korea | East China Sea | (Jeon et al., 2018) |
| Parviluciferaceae | *Parvilucifera multicavata* | *Alexandrium affine, A. catenella, Scrippsiella sp.,* Pyrophacus steinii | Asia | Korea | East China Sea | (Jeon and Park, 2020) |
| Parviluciferaceae | *Snorkelia* sp. Fosca 2016 | *Levanderina fissa* | Europe | Spain | Mediterranean Sea | (Reñé et al., 2017a) |
| Parviluciferaceae | *Snorkelia prorocentri* | *Prorocentrum fukuyoi, Levanderina fissa* | North America | Canada | Pacific Ocean | (Leander and Hoppenrath, 2008) |
| Parviluciferaceae | *Dinovorax pyriformis* | *Dinophysis sacculus* | Europe | Spain | Mediterranean Sea | (Reñé et al., 2017a) |
| Parviluciferaceae | *Tuberlatum coatsi* | *Alexandrium catenella, Scrippsiella sp.* | Asia | Korea | East China Sea | (Jeon and Park, 2019) |
| Parviluciferaceae | *Maranthos nigrum* | *Alexandrium affine, A. andersonii, A. catenella, A. mediterraneum, A. minutum, A. tamarense, A. taylorii, Gambierdiscus excentricus, Coolia tropicalis, Ostreopsis ovata, Heterocapsa triquetra, Kryptoperidinium foliaceum, Scrippsiella trochoidea, Prorocentrum rathymum, P. lima.* | Europe | Spain | NW Mediterranean Sea | (Reñé et al., 2021) |
| NAG01 | SPI agent | *Rana sphenocephala, R. sylvatica, R. heckscheri, R. clamitans, R. septentrionalis, R. pipens, R. sevosa, R. catesbeiana, capito, Pseudacris crucifer* | North America | USA | FL, GA, LA, MD, ME, MN, MS, NH, NY, OR, VA, WI | (Green et al., 2002; Davis et al., 2007; Jones et al., 2012; Landsberg et al., 2013; Isidoro-Ayza et al., 2017)) |
| Xcellidae | *Gadixcellia gadi* | *Gadus morhua* | Europe | Iceland | Atlantic Ocean | (Freeman *et al.*, 2017) |
| Xcellidae | *Gadixcellia* sp. | *Micromesistius poutassou* | Europe | Iceland | Atlantic Ocean | (Freeman et al., 2017) |
| Xcellidae | *Xcellia gobii* | *Acanthogobius flavimanus* | Asia | Japan | Pacific Ocean | (Freeman et al., 2017) |
| Xcellidae | *Xcellia lamelliphila* | *LImanda limanda, Lycodes spp., Macruronus*  *novaezelandiae, Merluccius gayi gayi, Trematomus spp.* | Europe | Scotland | North Sea | (Freeman et al., 2017) |
| Xcellidae | *Xcellia lamelliphila* | *Trematomus borchgrevinki, T. bernacchii, T. pennellii, T. nicolai* | Antartic | Antartic | Antartic sea | (Evans and Tupmongkol, 2014; Freeman et al., 2017) |
| Xcellidae | *Xcellia pleuronecti* | *Cleisthenes*  *herzensteini, Cleisthenes pinetorum, Glyptocephalus stelleri, Kareius bicoloratus, Hippoglossoides*  *dubius, Hippoglossoides elassodon, Liopsetta pinnifasciata, Platichthys stellatus, Parophrys vetulus,*  *Pseudopleuronectes obscurus, Pseudopleuronectes schrenki, Verasper moseri* | Asia | Japan | Pacific Ocean | (Freeman et al., 2017) |
|  | *Salmoxcellia vastator* | *Oncorhynchus mykiss, Salmo salar* | Europe | Norway | North Sea | (Karlsbakk et al., 2021) |

References:

Alacid, E., Reñé, A., Gallisai, R., Paloheimo, A., Garcés, E., and Kremp, A. (2020). Description of two new coexisting parasitoids of blooming dinoflagellates in the Baltic sea: *Parvilucifera catillosa* sp. nov. and *Parvilucifera* sp. (Perkinsea, Alveolata). *Harmful Algae* 100, 101944. doi:10.1016/j.hal.2020.101944.

Arzul, I., Chollet, B., Michel, J., Robert, M., Garcia, C., Joly, J.-P., et al. (2012). One *Perkinsus* species may hide another: characterization of *Perkinsus* species present in clam production areas of France. *Parasitology* 139, 1757–1771. doi:10.1017/S0031182012001047.

Azevedo, C. (1989). Fine structure of *Perkinsus atlanticus* n. sp. (Apicomplexa, Perkinsea) parasite of the clam *Ruditapes decussatus* from Portugal. *J. Parasitol.* 75, 627–35. doi:10.2307/3282915.

Cáceres-Martínez, J., Ortega, M. G., Vásquez-Yeomans, R., García, T. de J. P., Stokes, N. A., and Carnegie, R. B. (2012). Natural and cultured populations of the mangrove oyster *Saccostrea palmula* from Sinaloa, Mexico, infected by *Perkinsus marinus*. *J. Invertebr. Pathol.* 110, 321–325. doi:10.1016/j.jip.2012.03.019.

Casas, S. M., Grau, A., Reece, K. S., Apakupakul, K., Azevedo, C., and Villalba, A. (2004). *Perkinsus mediterraneus* n. sp., a protistan parasite of the European flat oyster *Ostrea edulis* from the Balearic Islands, Mediterranean Sea. *Dis. Aquat. Org.* 58, 231–244.

Choi, K.-S., and Park, K.-I. (2010). “Review on the protozoan parasite *Perkinsus olseni* (Lester and Davis 1981) infection in Asian waters,” in *Coastal Environmental and Ecosystem Issues of the East China Sea* (by TERRAPUB and Nagasaki University), 269–281.

Coss, C. A., Robledo, J. a. F., Ruiz, G. M., and Vasta, G. R. (2001). Description of *Perkinsus andrewsi* n. sp. isolated from the Baltic clam (*Macoma balthica*) by characterization of the ribosomal RNA locus, and development of a species-specific PCR-based diagnostic assay. *J. Eukaryot. Microbiol.* 48, 52–61. doi:https://doi.org/10.1111/j.1550-7408.2001.tb00415.x.

Cremonte, F., Balseiro, P., and Figueras, A. (2005). Occurrence of *Perkinsus olseni* (Protozoa: Apicomplexa) and other parasites in the venerid commercial clam *Pitar rostrata* from Uruguay, southwestern Atlantic coast. *Dis. Aquat. Org.* 64, 85–90. doi:10.3354/dao064085.

da Silva, P. M., Scardua, M. P., Vianna, R. T., Mendonça, R. C., Vieira, C. B., Dungan, C. F., et al. (2014). Two *Perkinsus* spp. infect *Crassostrea gasar* oysters from cultured and wild populations of the Rio São Francisco estuary, Sergipe, northeastern Brazil. *J. Invertebr. Pathol.* 119, 62–71. doi:10.1016/j.jip.2014.04.005.

da Silva, P. M., Vianna, R. T., Guertler, C., Ferreira, L. P., Santana, L. N., Fernández-Boo, S., et al. (2013). First report of the protozoan parasite *Perkinsus marinus* in South America, infecting mangrove oysters *Crassostrea rhizophorae* from the Paraíba River (NE, Brazil). *J. Invertebr. Pathol.* 113, 96–103. doi:10.1016/j.jip.2013.02.002.

Dantas Neto, M. P., Gesteira, T. C. V., Sabry, R. C., Feijó, R. G., Forte, J. M., Boehs, G., et al. (2016). First record of *Perkinsus chesapeaki* infecting *Crassostrea rhizophorae* in South America. *J. Invertebr. Pathol.* 141, 53–56. doi:10.1016/j.jip.2016.10.007.

Davis, A. K., Yabsley, M. J., Kevin Keel, M., and Maerz, J. C. (2007). Discovery of a novel alveolate pathogen affecting southern leopard frogs in Georgia: Description of the disease and host effects. *EcoHealth* 4, 310–317. doi:10.1007/s10393-007-0115-3.

Dungan, C. F., and Reece, K. S. (2006). *In vitro* propagation of two *Perkinsus* spp. parasites from Japanese Manila clams *Venerupis philippinarum* and description of *Perkinsus honshuensis* n. sp. *J. Eukaryot. Microbiol.* 53, 316–326. doi:https://doi.org/10.1111/j.1550-7408.2006.00120.x.

Dungan, C. F., Reece, K. S., Moss, J. A., Hamilton, R. M., and Diggles, B. K. (2007). *Perkinsus olseni in vitro* isolates from the New Zealand clam *Austrovenus stutchburyi*. *J. Eukaryot. Microbiol.* 54, 263–270. doi:https://doi.org/10.1111/j.1550-7408.2007.00265.x.

El Bour, M., Dellali, M., Boukef, I., Lakhal, F., Mraouna, R., El Hili, H. A., et al. (2012). First assessment of Perkinsosis and brown ring disease co-infection in *Ruditapes decussatus* in the North lake of Tunis (southern Mediterranean Sea). *J. Mar. Biol. Ass.* 92, 1579–1584. doi:10.1017/S0025315411001846.

Elandaloussi, L. M., Carrasco, N., Roque, A., Andree, K., and Dolores Furones, M. (2009). First record of *Perkinsus olseni*, a protozoan parasite infecting the commercial clam *Ruditapes decussatus* in Spanish Mediterranean waters. *J. Invertebr. Pathol.* 100, 50–53. doi:10.1016/j.jip.2008.09.004.

Enríquez-Espinoza, T., Grijalva-Chon, J., Castro-Longoria, R., and Ramos-Paredes, J. (2010). *Perkinsus marinus* in *Crassostrea gigas* in the Gulf of California. *Dis. Aquat. Org.* 89, 269–273. doi:10.3354/dao02199.

Evans, C. W., and Tupmongkol, K. (2014). X-cell disease in Antarctic fishes. *Polar Biol* 37, 1261–1269. doi:10.1007/s00300-014-1518-6.

Figueras, A., Fernández Robledo, J., and Novoa, B. (1992). Occurrence of haplosporidian and *Perkinsus*-like infections in carpet-shell clams, *Ruditapes decussatus* (Linnaeus 1758), of the Ria de Vigo (Galicia, NW Spain). *J. Shell. Res.* 11, 377–382.

Figueroa, R. I., Garcés, E., Massana, R., and Camp, J. (2008). Description, host-specificity, and strain selectivity of the dinoflagellate parasite *Parvilucifera sinerae* sp. nov. (Perkinsozoa). *Protist* 159, 563–578. doi:10.1016/j.protis.2008.05.003.

Freeman, M. A., Fuss, J., Kristmundsson, Á., Bjorbækmo, M. F. M., Mangot, J.-F., del Campo, J., et al. (2017). X-cells are globally distributed, genetically divergent fish parasites related to Perkinsids and Dinoflagellates. *Curr. Biol.* 27, 1645-1651.e3. doi:10.1016/j.cub.2017.04.045.

Garcés, E., and Hoppenrath, M. (12010). Ultrastructure of the intracellular parasite *Parvilucifera sinerae* (Alveolata, Myzozoa) infecting the marine toxic planktonic dinoflagellate *Alexandrium minutum* (Dinophyceae). *Harmful Algae* 10, 64–70. doi:10.1016/j.hal.2010.07.001.

Goggin, C. L., and Lester, R. J. G. (1995). *Perkinsus*, a protistan parasite of abalone in Australia: A review. *Mar. Freshwater Res.* 46, 639–646. doi:10.1071/mf9950639.

Green, D. E., Converse, K. A., and Schrader, A. K. (2002). Epizootiology of sixty-four amphibian morbidity and mortality events in the USA, 1996-2001. *Ann. N. Y. Acad. Sci.* 969, 323–339. doi:10.1111/j.1749-6632.2002.tb04400.x.

Gullian-Klanian, M., Herrera-Silveira, J., Rodríguez-Canul, R., and Aguirre-Macedo, L. (2008). Factors associated with the prevalence of *Perkinsus marinus* in *Crassostrea virginica* from the southern Gulf of Mexico. *Dis. Aquat. Org.* 79, 237–247. doi:10.3354/dao01910.

Hamaguchi, M., Suzuki, N., Usuki, H., and Ishioka, H. (1998). *Perkinsus* protozoan infection in short-necked clam *Tapes* (= *Ruditapes*) *philippinarum* in Japan. *Fish Pathol.* 33, 473–480. doi:https://doi.org/10.3147/jsfp.33.473.

Isidoro-Ayza, M., Lorch, J. M., Grear, D. A., Winzeler, M., Calhoun, D. L., and Barichivich, W. J. (2017). Pathogenic lineage of Perkinsea associated with mass mortality of frogs across the United States. *Sci. Rep.* 7, 10288. doi:10.1038/s41598-017-10456-1.

Itoh, N., Meyer, G., Tabata, A., Lowe, G., Abbott, C., and Johnson, S. (2013). Rediscovery of the Yesso scallop pathogen *Perkinsus qugwadi* in Canada, and development of PCR tests. *Dis. Aquat. Org.* 104, 83–91. doi:10.3354/dao02578.

Jeon, B. S., Nam, S. W., Kim, S., and Park, M. G. (2018). Revisiting the *Parvilucifera infectans* / *P. sinerae* (Alveolata, Perkinsozoa) species complex, two parasitoids of dinoflagellates. *Algae* 33, 1–19. doi:10.4490/algae.2018.33.3.6.

Jeon, B. S., and Park, M. G. (2019). *Tuberlatum coatsi* gen. n., sp. n. (Alveolata, Perkinsozoa), a new parasitoid with short germ tubes infecting marine dinoflagellates. *Protist* 170, 82–103. doi:10.1016/j.protis.2018.12.003.

Jeon, B. S., and Park, M. G. (2020). *Parvilucifera multicavata* sp. nov. (Alveolata, Perkinsozoa), a new parasitoid infecting marine dinoflagellates having abundant apertures on the sporangium. *Protist* 171, 125743. doi:10.1016/j.protis.2020.125743.

Johansson, M., Eiler, A., Tranvik, L., and Bertilsson, S. (2006). Distribution of the dinoflagellate parasite *Parvilucifera infectans* (Perkinsozoa) along the Swedish coast. *Aquat. Microb. Ecol.* 43, 289–302. doi:10.3354/ame043289.

Jones, M., Armién, A., Rothermel, B., and Pessier, A. (2012). Granulomatous myositis associated with a novel alveolate pathogen in an adult southern leopard frog (*Lithobates sphenocephalus*). *Dis. Aquat. Org.* 102, 163–167. doi:10.3354/dao02539.

Kang, H., Yang, H., Reece, K., Hong, H., Park, K., and Choi, K. (2016). First report of *Perkinsus honshuensis* in the variegated carpet shell clam *Ruditapes variegatus* in Korea. *Dis. Aquat. Org.* 122, 35–41. doi:10.3354/dao03063.

Karlsbakk, E., Nystøyl, C. F., Plarre, H., and Nylund, A. (2021). A novel protist parasite, *Salmoxcellia vastator* n. gen., n. sp. (Xcelliidae, Perkinsozoa), infecting farmed salmonids in Norway. *Parasites Vectors* 14, 431. doi:10.1186/s13071-021-04886-0.

Landsberg, J., Kiryu, Y., Tabuchi, M., Waltzek, T., Enge, K., Reintjes-Tolen, S., et al. (2013). Co-infection by alveolate parasites and frog virus 3-like ranavirus during an amphibian larval mortality event in Florida, USA. *Dis. Aquat. Org.* 105, 89–99. doi:10.3354/dao02625.

Lepelletier, F., Karpov, S. A., Le Panse, S., Bigeard, E., Skovgaard, A., Jeanthon, C., et al. (2014). *Parvilucifera rostrata* sp. nov. (Perkinsozoa), a novel parasitoid that infects planktonic dinoflagellates. *Protist* 165, 31–49. doi:10.1016/j.protis.2013.09.005.

Marquis, N. D., Bishop, T. J., Record, N. R., Countway, P. D., and Fernández Robledo, J. A. (2020). A qPCR-based survey of *Haplosporidium nelsoni* and *Perkinsus* spp. in the eastern oyster, *Crassostrea virginica* in Maine, USA. *Pathogens* 9, 256. doi:10.3390/pathogens9040256.

Marsh, A., Gauthier, J., and Vasta, G. (1995). A semiquantitative PCR assay for assessing *Perkinsus marinus* infections in the Eastern Oyster, *Crassostrea virginica*. *J. Parasitol.* 81, 577–83. doi:10.2307/3283856.

Mclaughlin, S. M., Tall, B. D., Shaheen, A., Elsayed, E. E., and Faisal, M. (2000). Zoosporulation of a new *Perkinsus* species isolated from the gills of the softshell clam *Mya arenaria*. *Parasite* 7, 115–122. doi:10.1051/parasite/2000072115.

Moss, J. A., Xiao, J., Dungan, C. F., and Reece, K. S. (2008). Description of *Perkinsus beihaiensis* n. sp., a new *Perkinsus* sp. parasite in oysters of southern China. *J. Eukaryot. Microbiol.* 55, 117–130. doi:10.1111/j.1550-7408.2008.00314.x.

Murrell, A., Kleeman, S. N., Barker, S. C., and Lester, R. J. G. (2002). Synonymy of *Perkinsus olseni* Lester & Davis, 1981 and *Perkinsus atlanticus* Azevedo, 1989 and an update on the phylogenetic position of the genus *Perkinsus*. *Bull. Eur. Ass. Fish Pathol.* 22, 258–265.

Norén, F., Moestrup, Ø., and Rehnstam-Holm, A.-S. (1999). *Parvilucifera infectans* Norén et Moestrup gen. et sp. nov. (Perkinsozoa phylum nov.): a parasitic flagellate capable of killing toxic microalgae. *Eur. J. Protistol.* 35, 233–254. doi:10.1016/S0932-4739(99)80001-7.

Park, K.-I., Ngo, T. T. T., Choi, S.-D., Cho, M., and Choi, K.-S. (2006). Occurrence of *Perkinsus olseni* in the Venus clam *Protothaca jedoensis* in Korean waters. *J. Invertebr. Pathol.* 93, 81–87. doi:10.1016/j.jip.2006.04.007.

Park, K.-I., Tsutsumi, H., Hong, J.-S., and Choi, K.-S. (2008). Pathology survey of the short-neck clam *Ruditapes philippinarum* occurring on sandy tidal flats along the coast of Ariake Bay, Kyushu, Japan. *J. Invertebr. Pathol.* 99, 212–219. doi:10.1016/j.jip.2008.06.004.

Pinho Ferreira, L., Sabry, R. C., da Silva, P. M., Gesteira, T. C. V., de Souza Romão, L., Paz, M. P., et al. (2015). First report of *Perkinsus beihaiensis* in wild clams *Anomalocardia brasiliana* (Bivalvia: Veneridae) in Brazil. *Exp. Parasitol.* 150, 67–70. doi:10.1016/j.exppara.2014.07.012.

Pretto, T., Zambon, M., Civettini, M., Caburlotto, G., Boffo, L., Rossetti, E., et al. (2014). Massive mortality in Manila clams (*Ruditapes philippinarum*) farmed in the lagoon of Venice, caused by *Perkinsus olseni*. *Bull. Eur. Ass. Fish Pathol.* 34, 43.

Ramilo, A., Carrasco, N., Reece, K. S., Valencia, J. M., Grau, A., Aceituno, P., et al. (2015). Update of information on perkinsosis in NW Mediterranean coast: Identification of *Perkinsus* spp. (Protista) in new locations and hosts. *J. Invertebr. Pathol.* 125, 37–41. doi:10.1016/j.jip.2014.12.008.

Reece, K., Bushek, D., Hudson, K., and Graves, J. (2001). Geographic distribution of *Perkinsus marinus* genetic strains along the Atlantic and Gulf Coasts of the USA. *Mar. Biol.* 139, 1047–1055. doi:10.1007/s002270100657.

Reece, K., Dungan, C., and Burreson, E. (2008). Molecular epizootiology of *Perkinsus marinus* and *P. chesapeaki* infections among wild oysters and clams in Chesapeake Bay, USA. *Dis. Aquat. Org.* 82, 237–248. doi:10.3354/dao01997.

Reñé, A., Alacid, E., Ferrera, I., and Garcés, E. (2017a). Evolutionary trends of Perkinsozoa (Alveolata) characters based on observations of two new genera of parasitoids of dinoflagellates, *Dinovorax* gen. nov. and *Snorkelia* gen. nov. *Front. Microbiol.* 8. doi:10.3389/fmicb.2017.01594.

Reñé, A., Alacid, E., Figueroa, R. I., Rodríguez, F., and Garcés, E. (2017b). Life-cycle, ultrastructure, and phylogeny of *Parvilucifera corolla* sp. nov. (Alveolata, Perkinsozoa), a parasitoid of dinoflagellates. *Eur. J. Protistol.* 58, 9–25. doi:10.1016/j.ejop.2016.11.006.

Reñé, A., Alacid, E., Gallisai, R., Chambouvet, A., Fernández‐Valero, A. D., and Garces, E. (2021). New Perkinsea parasitoids of dinoflagellates distantly related to the Parviluciferaceae members. *Front. Microbiol.* 12, 2199. doi:10.3389/fmicb.2021.701196.

Rodríguez, F., and Figueroa, R. I. (2020). Confirmation of the wide host range of *Parvilucifera corolla* (Alveolata, Perkinsozoa). *Eur. J. Protistol.*, 125690. doi:10.1016/j.ejop.2020.125690.

Sabry, R. C., Rosa, R. D., Magalhães, A. R. M., Barracco, M. A., and Gesteira, T. C. V. (2009). First report of *Perkinsus* sp. infecting mangrove oysters *Crassostrea rhizophorae* from the Brazilian coast. *Dis. Aquat. Org.* 88, 13–23.

Sanil, N. K., Vijayan, K. K., Kripa, V., and Mohamed, K. S. (2010). Occurrence of the protozoan parasite, *Perkinsus olseni* in the wild and farmed Pearl Oyster, *Pinctada fucata* (Gould) from the Southeast coast of India. *Aquaculture* 299, 8–14. doi:10.1016/j.aquaculture.2009.12.007.

Sanil, N., Suja, G., Lijo, J., and Vijayan, K. (2012). First report of *Perkinsus beihaiensis* in *Crassostrea madrasensis* from the Indian subcontinent. *Dis. Aquat. Org.* 98, 209–220. doi:10.3354/dao02440.

Shamal, P. (2018). *Perkinsus olseni* in the short neck yellow clam, *Paphia malabarica* (Chemnitz, 1782) from the southwest coast of India. *J. Invertebr. Pathol.* 159, 113–120.

Sheppard, B., and Phillips, A. (2008). *Perkinsus olseni* detected in Vietnamese aquacultured reef clams *Tridacna crocea* imported to the USA, following a mortality event. *Dis. Aquat. Org.* 79, 229–235. doi:10.3354/dao01888.

Wu, S., Wang, C., Lin, X., Wang, Z., Li, X., Liu, J., et al. (2011). Infection prevalence and phylogenetic analysis of *Perkinsus olseni* in *Ruditapes philippinarum* from East China. *Dis. Aquat. Org.* 96, 55–60. doi:10.3354/dao02353.
